# Emergency Venovenous Extracorporeal Membrane Oxygenation–Supported Repair of Complex Right Main Bronchus and Pulmonary Artery Rupture

**DOI:** 10.1016/j.atssr.2025.11.010

**Published:** 2025-12-05

**Authors:** Valentine Petit, Emma Pamies, Antoine Sion, Victor Lestrade, Frederic Wallyn, Marie Boulo, Delphine Garrigue, Nicolas Venissac, Julien De Wolf

**Affiliations:** 1 Department of Thoracic Surgery, Lille University Hospital, France; 2 Department of Anesthesia and Critical Care, Lille University Hospital, France; 3 Department of Pneumology, Lille University Hospital, France; 4 Surgical Critical Care Unit, Department of Anesthesiology, Critical Care and Perioperative Medicine, Lille University Hospital, France

## Abstract

Traumatic rupture of the right main bronchus with pulmonary artery injury is exceptionally rare and often fatal when pneumonectomy is required. An 18-year-old man presented with bronchial transection and pulmonary artery rupture after chest trauma. Venovenous extracorporeal membrane oxygenation enabled oxygenation during controlled thoracotomy. A parenchyma-sparing strategy was adopted: right upper lobectomy, pulmonary artery repair, and end-to-end bronchial anastomosis, avoiding pneumonectomy. Secondary bronchial dehiscence with bronchovascular fistula was successfully managed by omental flap repair. This case demonstrates that early recognition, extracorporeal membrane oxygenation support, and lung-preserving reconstruction are key to survival in catastrophic bronchovascular trauma.

Traumatic rupture of the right main bronchus (RMB) is rare and highly lethal,^1^^,^^2^ particularly when associated with vascular injury. In such cases, emergency pneumonectomy has historically been the default strategy, but outcomes are extremely poor, with mortality rates approaching 100% after blunt trauma. The causes of death include acute right ventricular failure, refractory hypoxemia, and massive hemorrhage. In contrast, parenchyma-sparing procedures such as lobectomy or bronchovascular reconstruction can significantly improve survival by preserving lung function and reducing hemodynamic strain. Recent advances in extracorporeal membrane oxygenation (ECMO) now allow perioperative stabilization of severely hypoxemic trauma patients, enabling controlled thoracotomy and complex repair. We report the case of an 18-year-old motorcyclist with combined RMB and pulmonary artery rupture, successfully managed with emergency venovenous extracorporeal membrane oxygenation (VV-ECMO) support and parenchyma-sparing reconstruction. Avoidance of pneumonectomy was decisive for survival.

An 18-year-old male individual was admitted after a motorcycle–truck collision with severe right-sided chest trauma. On arrival, he was tachypneic, hypoxemic, and had absent right breath sounds. Chest radiograph confirmed complete right pneumothorax with persistent collapse despite 2 chest drains (Figure 1). Bronchoscopy revealed a drain protruding into the RMB, confirming transection. Attempts at selective intubation precipitated profound desaturation (<20% SpO_2_). The patient was transferred to the operating room, where an emergency femorojugular VV-ECMO was initiated, rapidly restoring oxygenation and hemodynamic stability.

**Figure 1:**
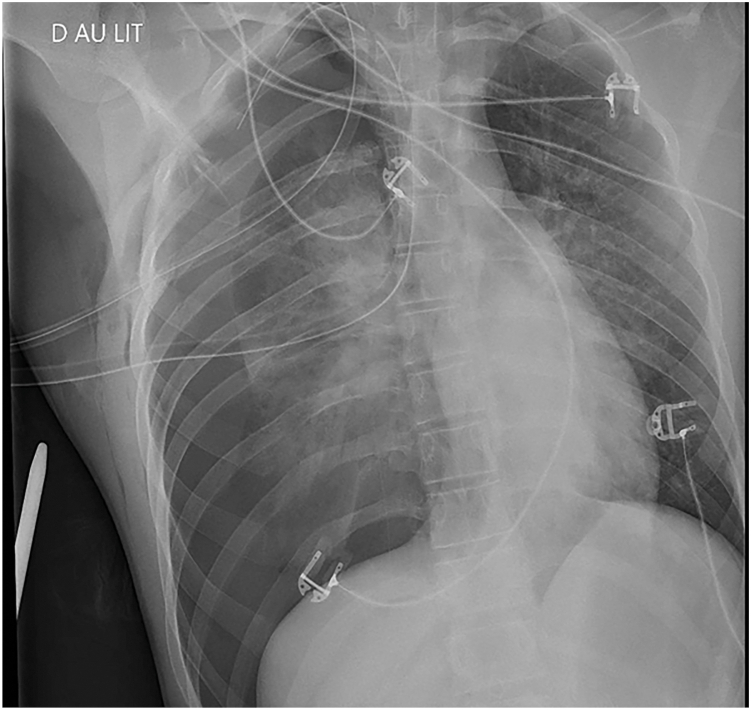
Initial chest radiograph showing persistent right pneumothorax despite placement of two chest drains.

The patient was placed in left lateral decubitus and underwent right posterolateral thoracotomy. Findings included complete RMB transection extending to the bronchus intermedius, destruction of the right upper lobe, hemopneumothorax, and a contained rupture of the mediastinal pulmonary artery branch. A pneumonectomy was initially considered but rejected due to prohibitive mortality. Instead, a lung-preserving approach was adopted. Wide pericardiotomy provided control of the pulmonary artery and veins. A right upper lobectomy was performed, the mediastinal pulmonary artery branch was repaired with running 5-0 polypropylene, and an end-to-end anastomosis reconnected the RMB to the bilobar bronchial stump. Two chest drains were placed, bilobar reexpansion confirmed, and VV-ECMO successfully discontinued intraoperatively.

The postoperative course was complicated by pneumonia leading to failed extubation and tracheostomy. Four weeks later, bronchoscopy revealed partial anterior bronchial dehiscence with bronchovascular fistula. Rethoracotomy was performed with bronchial repair and interposition of a vascularized omental flap. Recovery was uneventful thereafter. At follow-up, bronchoscopy demonstrated complete healing of the bronchial anastomosis and the chest radiograph showed preserved bilobar function (Figure 2).

**Figure 2:**
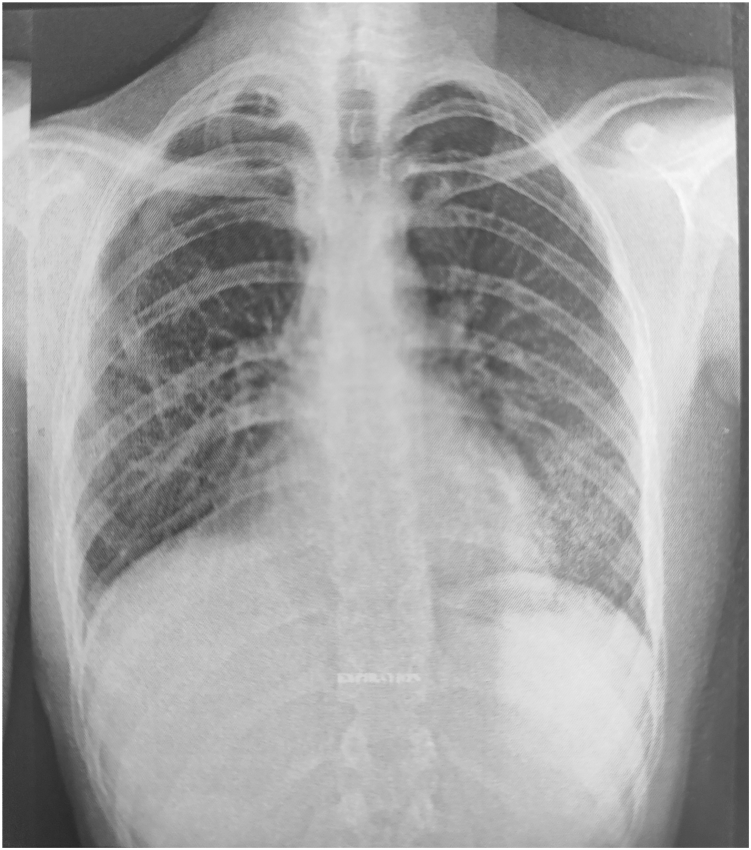
Follow-up chest radiograph at 2 months postoperatively demonstrating complete expansion of the right lung with excellent radiologic outcome.

## Comment

Traumatic bronchovascular injuries remain among the most lethal presentations in thoracic surgery. Although rare, rupture of the right main bronchus combined with pulmonary artery injury is almost uniformly fatal when treated by emergency pneumonectomy. Several studies have consistently reported mortality rates^3^^,^^4^ between 60% and nearly 100% after traumatic pneumonectomy. The principal mechanisms include acute right ventricular failure due to abrupt increases in pulmonary vascular resistance, refractory hypoxemia from loss of functional lung tissue, and overwhelming sepsis. Symbas and colleagues^3^ documented near-universal mortality in blunt airway trauma managed with pneumonectomy, while Angelillo Mackinlay and coworkers^4^ described pneumonectomy as a “continuing lethal operation” in thoracic trauma. These observations highlight the futility of pneumonectomy in this context.

Whenever possible, lung-preserving approaches offer significantly better outcomes. Parenchyma-sparing resections reduce right heart strain, preserve gas exchange, and improve postoperative recovery. Even in complex scenarios involving complete airway transection and vascular disruption, meticulous reconstruction can achieve survival if performed in an experienced center. Balci and associates^5^ demonstrated superior survival with parenchyma-sparing resections compared with pneumonectomy for posttraumatic bronchial injuries. Similarly, Koletsis and colleagues^6^ confirmed that lobectomy or sleeve resections, when feasible, are associated with favorable outcomes. These reports support the principle that, in airway trauma, resection should be limited to the nonviable segment and reconstruction attempted whenever technically possible.

An additional lesson from this case concerns the pivotal role of ECMO in enabling definitive repair. Traditional ventilation often fails in complete mainstem bronchus disruption, as selective intubation is usually impossible. In our patient, repeated attempts caused profound hypoxemia and cardiac arrest. Rapid femorojugular VV-ECMO restored oxygenation and provided a bridge to thoracotomy under controlled conditions. Compared with cardiopulmonary bypass, ECMO is faster to deploy, requires less anticoagulation, and is thus more suitable in trauma. Although prolonged postoperative ECMO might have mitigated lung injury, it was discontinued at surgery’s end owing to stable gas exchange and hemodynamics. Full injury assessment was still pending, and continued ECMO would have complicated transfers and increased bleeding or cannula-related risks. Early weaning therefore represented the safest balance between respiratory support and trauma management.

A further technical consideration is protection of suture lines when airway and vascular repairs coexist. At the initial operation, no intercostal muscle flap was placed because the field was severely distorted by chest wall hematoma, rib fractures, and mediastinal exposure. In retrospect, buttressing might have offered additional protection. Four weeks later, partial bronchial dehiscence with bronchovascular fistula required reoperation, during which an omental flap was interposed between bronchial and arterial repairs. Previous authors, including Mathisen and Grillo,^7^ have emphasized the value of vascularized tissue buttressing in such settings. In our patient, this delayed reinforcement proved decisive for long-term healing.

This case underscores 3 central lessons for managing extreme thoracic trauma. First, emergency pneumonectomy should be avoided whenever possible, given its prohibitive lethality. Second, parenchyma-sparing techniques, though technically demanding, can preserve function and survival. Third, VV-ECMO provides a critical bridge to stabilization, transforming otherwise nonsurvivable injuries into potentially manageable situations. Importantly, this case also represents a rare departure from conventional “damage control” principles, which usually favor rapid, simple interventions in unstable trauma patients. Here, only a complex, definitive repair—made feasible by ECMO—ensured survival. These insights reinforce the need for experienced surgical teams, rapid access to extracorporeal support, and a bias toward parenchyma preservation in catastrophic bronchovascular trauma.

Emergency pneumonectomy for thoracic trauma carries prohibitive mortality. Whenever technically feasible, parenchyma-sparing procedures such as lobectomy or complex bronchovascular reconstruction should be prioritized. In this case, survival was achieved by combining VV-ECMO as a bridge to oxygenation with meticulous reconstruction of both airway and vasculature. This strategy preserved bilobar function and prevented the fatal complications typically associated with pneumonectomy. The case also illustrates a rare exception to the damage control paradigm: here, immediate and complex repair, rather than a rapid radical procedure, was the only life-saving option. Early recognition of the futility of pneumonectomy and timely use of ECMO can transform outcomes in otherwise catastrophic bronchovascular trauma.

## References

[1] Kiser A.C., O'Brien S.M., Detterbeck F.C. (2001). Blunt tracheobronchial injuries: treatment and outcomes. Ann Thorac Surg.

[2] Bertelsen S., Howitz P. (1972). Injuries of the trachea and bronchi. Thorax.

[3] Symbas P.N., Justicz A.G., Ricketts R.R. (1992). Rupture of the airways from blunt trauma: treatment of complex injuries. Ann Thorac Surg.

[4] Angelillo Mackinlay T.A., Lyons G.A., Chitwood W.R. (1995). Acute traumatic pneumonectomy: a continuing lethal operation. Ann Thorac Surg.

[5] Balci A.E., Eren N., Eren S., Ulku R. (2002). Surgical treatment of post-traumatic tracheobronchial injuries: 14-year experience. Eur J Cardiothorac Surg.

[6] Koletsis E.N., Prokakis C., Baltayiannis N. (2014). Surgical management of bronchial rupture due to blunt chest trauma: lung parenchyma-sparing techniques improve outcome. Eur J Cardiothorac Surg.

[7] Mathisen D.J., Grillo H.C. (1992). Omental pedicle wrap after sleeve resection of the airway. Ann Thorac Surg.

